# A Large Board Pin in the Right Main Bronchus: A Case Report With Review of Literature

**DOI:** 10.7759/cureus.60350

**Published:** 2024-05-15

**Authors:** Dinesh Kumar Sathanantham, Vinitha Vishwambharam Nair, Paras Ramesh lalwani, K K Athish, Sravani Bhavanam, Bejoi Mathew, Jayakumar Thanathu Krishnan Nair

**Affiliations:** 1 Cardiovascular and Thoracic Surgery, Government Medical College Kottayam, Kottayam, IND; 2 General Surgery, Sri Devaraj Urs Medical College, Sri Devaraj Urs Academy of Higher Education and Research, Chennai, IND; 3 Internal Medicine, Sri Devaraj Urs Medical College, Sri Devaraj Urs Academy of Higher Education and Research, Kolar, IND

**Keywords:** asymptomatic, rigid bronchoscopy, board pin aspiration, pediatric airway safety, bronchoscopy, aspiration, airway foreign body

## Abstract

Foreign body (FB) aspiration is one of the most common emergency scenarios in cardiothoracic surgery and ENT unit consultations. We present the case of a 16-year-old male student who inadvertently ingested board pins while enjoying leftover savory. Despite the initial shock, he promptly sought evaluation at the local primary care facility. Remarkably, he remained largely asymptomatic. A subsequent chest radiograph revealed a radiopaque FB lodged in the right main bronchus. Employing a rigid bronchoscope, we successfully extracted the FB, obviating the need for open surgical intervention. What sets this case apart is the unusual combination of a large FB aspiration with minimal symptoms and the absence of internal injury during retrieval.

## Introduction

Foreign body (FB) aspiration refers to the inhalation of any object into the tracheobronchial tree. It can be a very deleterious situation and occasionally fatal, but adequate and prompt treatment is linked with low rates of mortality and morbidity [1]. Although this condition is commonly noticed in children, adults with aspirated FBs are a routine emergency in the cardiothoracic and otolaryngology units [2]. Clinical presentation can vary by location of FB, from asymptomatic to coughing or dyspnea. In certain scenarios, the patient develops sudden coughing, retching, and vomiting when an FB penetrates, and upright posture is more prone to inadvertent inhalation of FBs into the tracheobronchial tree or airway. Also, the aspiration of foreign sharp objects, such as needles or pins, is acknowledged as a critical medical emergency that poses a threat to life. Thus, its extraction is an exhaustive undertaking [3]. In contrast, pin aspiration generally presents either asymptomatically or can present with a cough and/or breathlessness despite a distinct history [4]. As anatomically, the right bronchus is shorter and wider, the FBs in tracheobronchial aspiration are more likely to lodge here [5]. Emergency rigid endoscopy is generally employed to extract the aspirated FB from the mainstem bronchus; however, a few authors have recently opted for flexible bronchoscopy as an alternative [5]. A clear history of FB aspiration with clinical signs and symptoms is sufficient to warrant bronchoscopy. Also, it is crucial to distinguish an airway FB aspiration from a gastrointestinal FB in other cases.

In this case, we report board pin aspiration by an adolescent and its further management with emergency bronchoscopic removal.

## Case presentation

**Figure 1:**
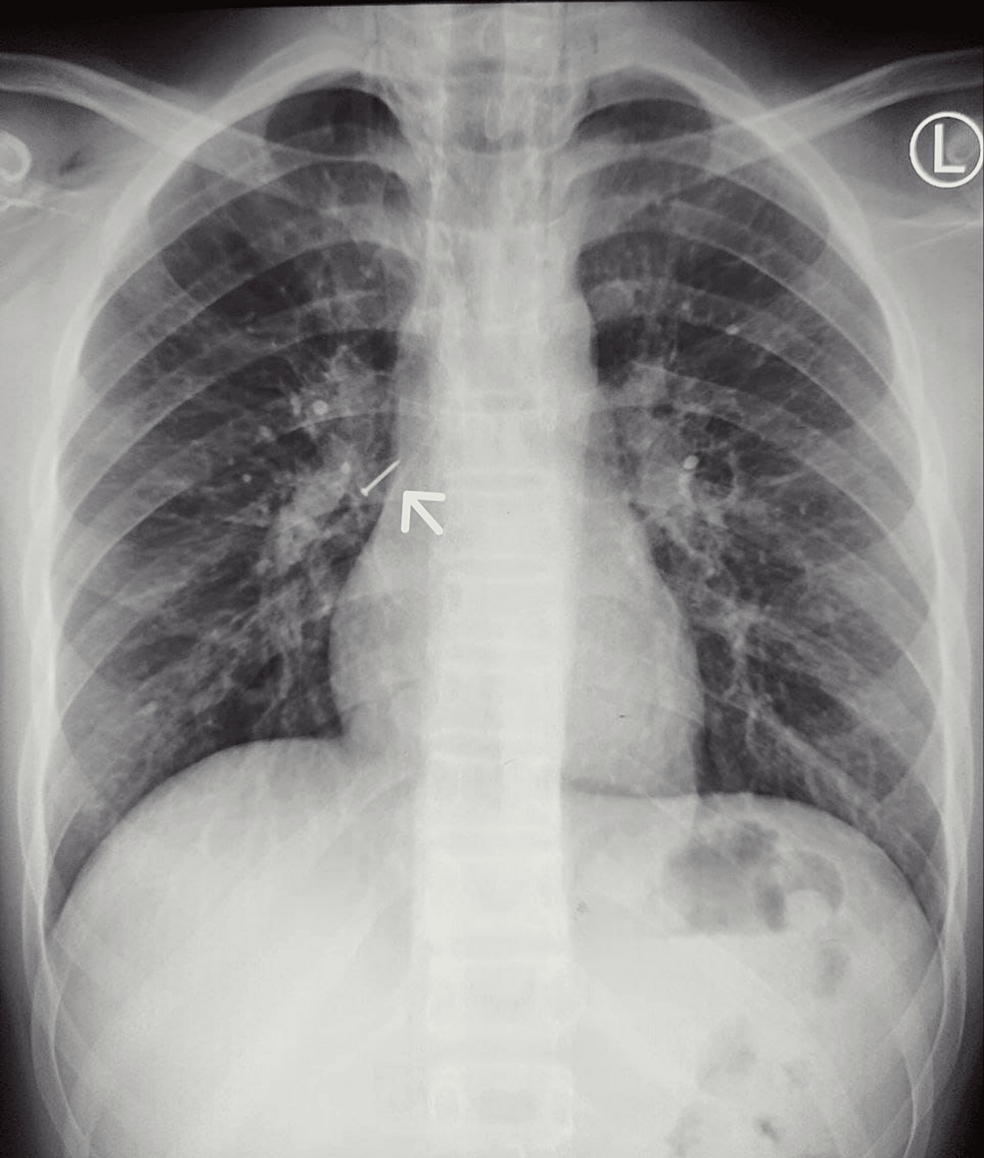
Chest radiograph showing a clear-cut radiopacity over the right main bronchus, suggestive of a pin (white arrow)

A 16-year-old schoolboy presented to the ER with anxiety after aspirating an FB. He reported noticing board pins among the leftover savory that he was consuming while sitting in an upright position. He had no history of breathlessness, chest pain, coughing, expectoration, or vomiting. On physical examination, the patient was alert and receptive, with a SpO2 of 98% at room air, a pulse rate of 83 beats/minute, a respiratory rate of 17 breaths/minute, and a blood pressure of 110/74 mmHg. A chest radiograph revealed a clear-cut radiopaque body over the right main bronchus, suggestive of a pin (Figure 1).

**Figure 2:**
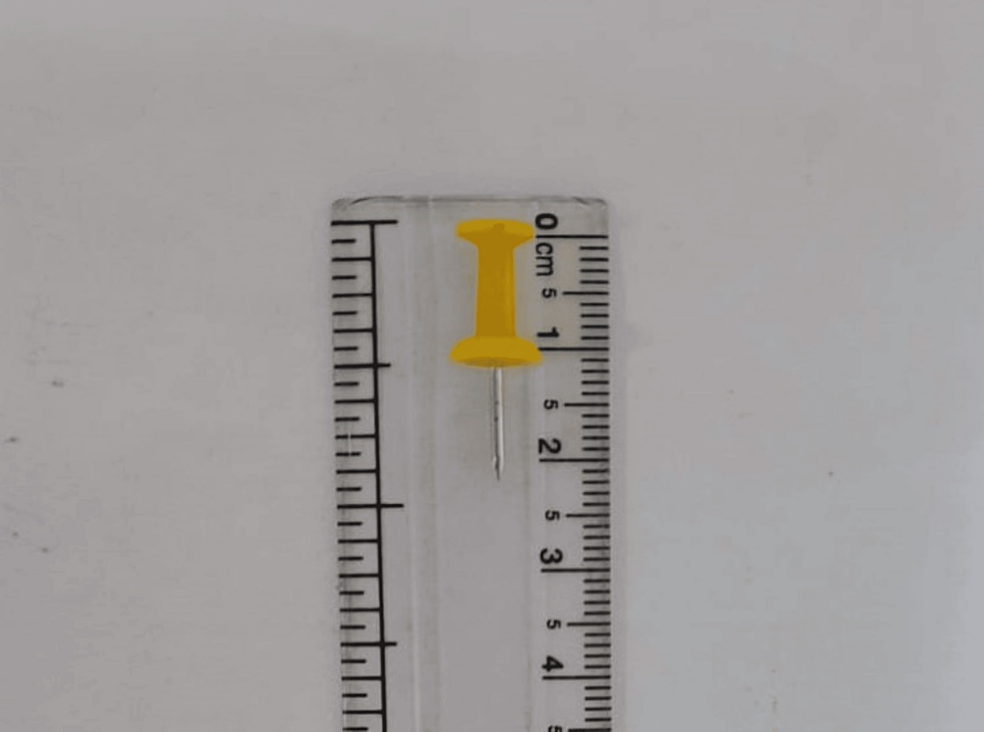
Retrieved board pin (approximately 2.2 cm)

Patient bystanders were informed about the necessity of emergency retrieval due to a strong suspicion of aspiration of a relatively large FB and the associated risk of life-threatening obstruction of the airway. Upon obtaining consent, an emergency rigid bronchoscopy was performed to promptly and securely remove the FB. The FB has stuck, partially obstructing the right mainstem bronchus. With the aid of a rigid bronchoscopy approach under general anesthesia, FB was located, grasped, and removed with crocodile forceps, and a board pin was extracted (Figure 2).

**Figure 3:**
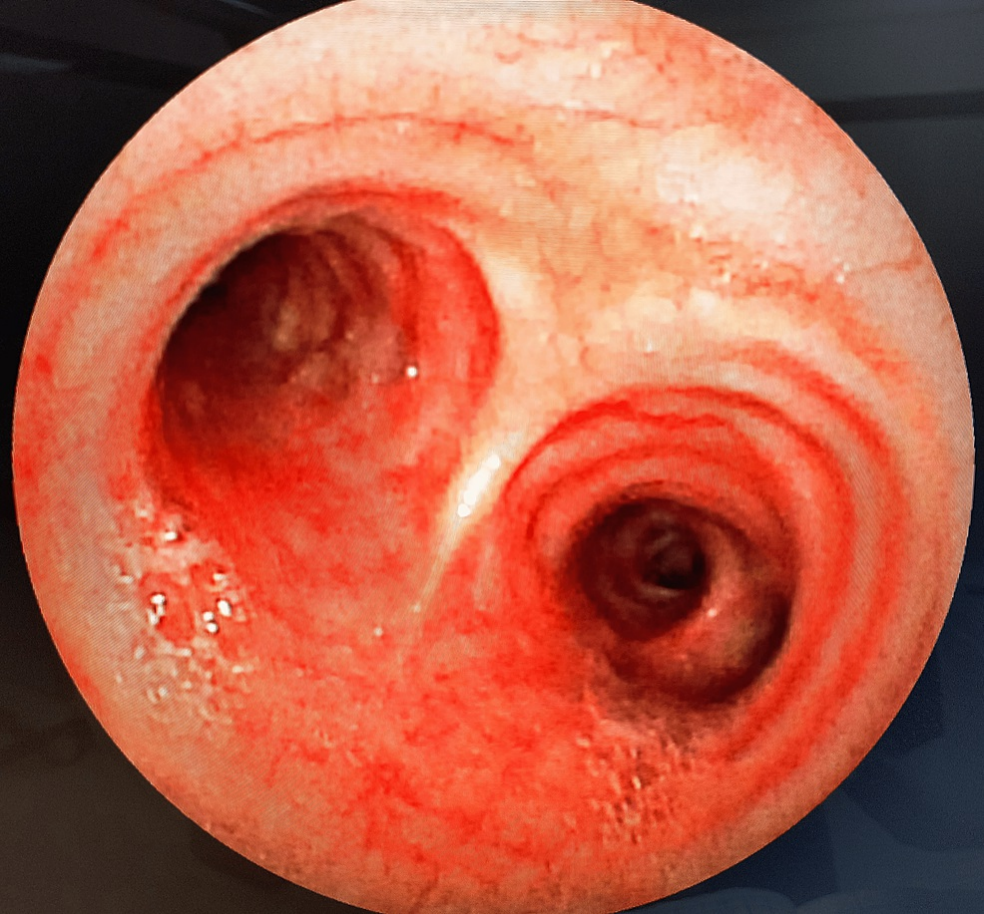
Post-FB retrieval check bronchoscopy showing a patent airway in the right main bronchus with no internal injury FB, foreign body

**Figure 4:**
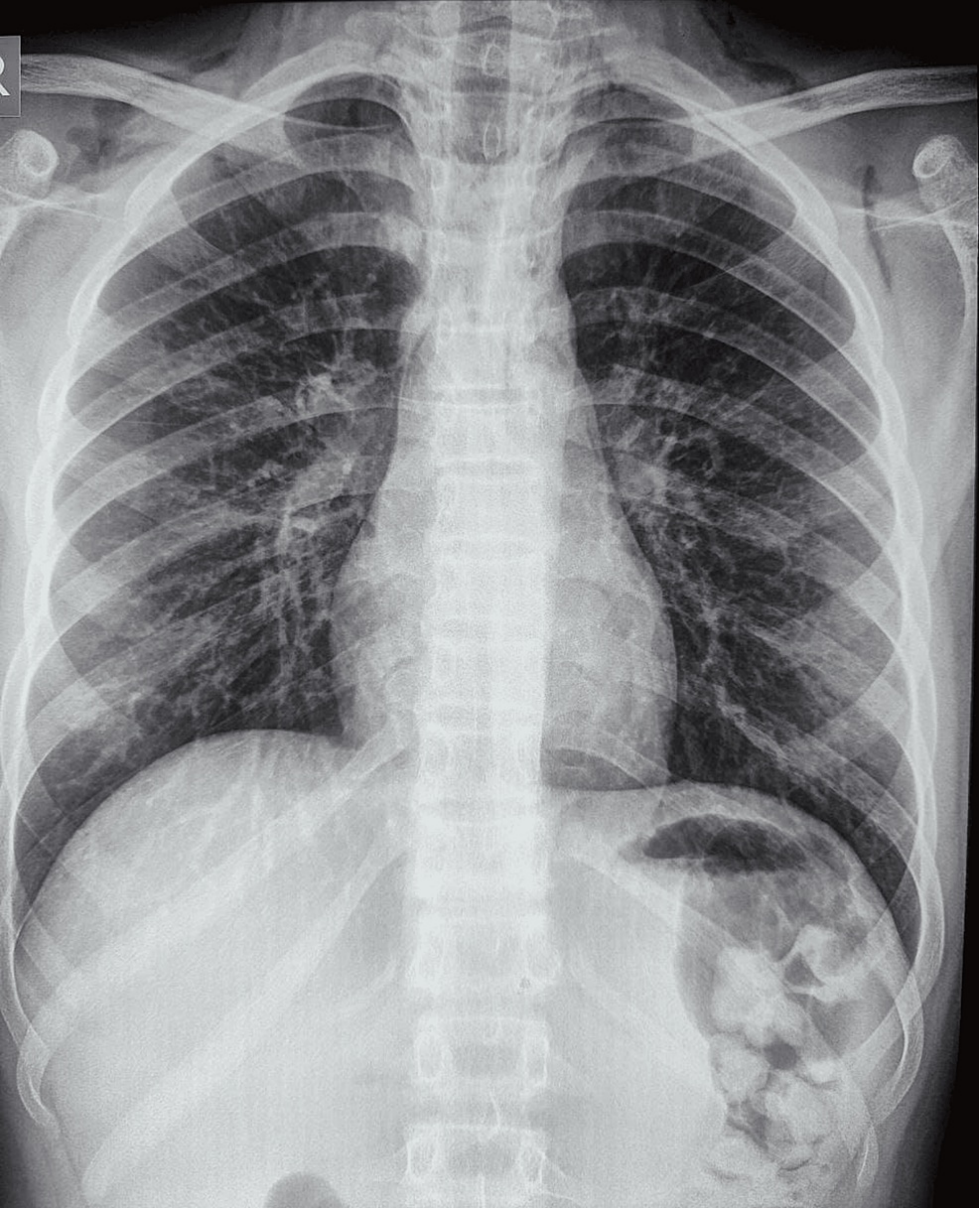
Post-rigid bronchoscopy chest radiograph demonstrating the successful retrieval of the board pin

Following the removal of the pin, airway inspection with a check bronchoscopy revealed a patent airway in the right main bronchus and distal segments (Figure 3). The procedure was performed by a combined team consisting of a cardiothoracic surgeon, pulmonologist, and anesthesiologist. A repeat chest radiograph taken after three and 12 hours was normal (Figure 4); the patient was admitted for overnight observation; a pulmonology opinion was taken; and the child was discharged the following day. At the follow-up visit, the child was doing well.

## Discussion

FBs, either accidental ingestion or aspiration, are very common but grossly preventable problems that are more widespread among children aged three months to six years [6]. The majority of FBs in the pediatric age group consist of toys, food particles, or others, like various nuts, seeds, or button electronic batteries [7].

Alternatively, in later stages of life, it may arise as a result of cognitive impairment or psychiatric conditions, as identified by Reilly and Carr, who attributed this occurrence to factors that encompass insufficient regulation of hand-to-mouth coordination, oral exploration of objects, prolonged oral phase, communication difficulties, and compromised protective mechanisms [8]. In children, it is uncommon to find a pin for an FB aspiration, although turban pin aspiration is frequently reported in Muslim women [7,9]. However, in this case, the patient is a healthy schoolboy aged 16 who aspirated the board pin accidentally while consuming savory.

The aspirated FB can lodge anywhere between the terminal bronchioles and the laryngeal inlet. Age and the patient’s position during aspiration determine the location of the FB in both bronchi. Up to the age of 15, the mainstem bronchi’s angle made with the trachea is identical, leading to the same frequency of aspiration of FBs in either bronchus. After 15 years, the angle between the two bronchus changes owing to differential growth and development. Hence, the right bronchus aligns itself more closely with the trachea, creating a reasonably straight path between the larynx and bronchus [10]. In our case, the lodgment was in the anatomically more common right main stem bronchus.

For an attending doctor, ingestion of sharp items by accident is typically treated as an emergency because of the high possibility of the objects piercing the aerodigestive tract and causing life-threatening complications. However, the symptoms can range from potentially fatal acute asphyxiation to partial airway obstruction causing coughing, breathing difficulty, and choking spells [2]. Children with FB aspiration usually present with symptoms of coughing, wheezing, dyspnea, stridor, or cyanosis [11]. Coughing or choking spells require immediate medical attention. FB’s motion and irritation of the mucosa within the tracheobronchial tree are the main indications of FB aspiration. The cough ceases after the FB settles in the bronchus [5]. In asymptomatic patients, while symptoms may offer diagnostic clues, relying on a thorough history and imaging, as in this case, can often provide ample information.

Delayed removal of intrabronchial FB causes major complications like obstructive emphysema, pulmonary abscess, pneumomediastinum, bronchiectasis, and recurrent pneumonia [12].

The evaluation of a suspected FB aspiration patient includes a plain chest radiograph with anteroposterior and lateral radiographs [13]. Radiographs can easily diagnose radiopaque FB (coins and metallic toys), but they constitute a minor percentage of aspirated FBs, with the majority being radiodense (food particles and nuts); hence, a negative film does not exclude aspiration [14]. Other radiographic features suggestive of an aspirated FB are air trapping (a ball valve permits air to enter the bronchus but occludes it during expiration), atelectasis (complete or partial obstruction of the bronchus), and obstructive pneumonia [15].

Initial management of FB aspiration or ingestion starts with airway management, and the level of care is based on the clinical presentation. In high-risk fatal asphyxiation cases, immediate endotracheal intubation, or in circumstances where attempts at ventilation have demonstrated failure, an emergent tracheostomy or cricothyrotomy is necessary to secure the airway. In non-asphyxiating cases, supplemental oxygen might be sufficient. Once the airway has been secured, imaging should be considered to localize the FB.

Based on existing data, since most pins are located close to the hilum, they can be retrieved via bronchoscopy. We speculate this could be because of the large plastic pin heads, which prevent them from going any farther [9].

The following are the observed concerns during the bronchoscopic removal of aspirated FB from the airway: hemorrhage, laryngeal edema, cardiac arrest, pneumomediastinum, and pneumothorax [5].

Gustav Killian of Freiburg first attempted rigid bronchoscopy in 1887 to extract a pork bone that a farmer had aspirated while consuming soup. Experts prefer rigid bronchoscopy, which continues to be the procedure of choice for FB retrieval as it facilitates securing the airway quickly [16]. When it comes to removing tracheobronchial FB, rigid bronchoscopy succeeds over flexible bronchoscopy because it can be done under general anesthesia, displays better airway control, has better visualization, and is easier to manipulate. However, patient-related factors and operator skills also play a role in determining the successful outcome of the procedure. The prevalence of FB aspiration-related mortality varies from 1% to 12% depending on the variety of FB aspirated; sharp items have the highest incidence [17].

The most notable aspect of our report is the typical yet uncommon presentation of our case, which was nearly asymptomatic. The 16-year-old student accidentally swallowed a board pin while eating savory food in an upright position. Remarkably, the pin passed through the respiratory tract vertically instead of horizontally, thus avoiding laceration of the trachealis muscle. Ultimately, through rigid bronchoscopy, we successfully extracted the FB in its entirety without causing any damage to the trachea, thereby eliminating the need for flexible bronchoscopy or thoracotomy.

We recommend a multidisciplinary approach involving pulmonologists, radiologists, cardiothoracic surgeons, otolaryngologists, and anesthesiologists for the management of FBs in children, spanning from diagnosis to treatment. Prompt evaluation for diagnosis, FB localization, and treatment considerations, such as bronchoscopy (rigid vs. flexible) or open surgical options like bronchotomy, are crucial aspects of FB extraction management.

## Conclusions

FB aspiration is one of the most common surgical emergencies in the ER. In this report, we highlight an atypical case presentation from an epidemiological perspective, managed through a conventional treatment approach involving rigid bronchoscopy. We wish to emphasize that rigid bronchoscopy remains a standard modality for FB retrieval and should not be overlooked despite the advancements in medicine toward flexible bronchoscopy.
